# Retraction: Characterization of Vascular plant One-Zinc finger (VOZ) in soybean (*Glycine max* and *Glycine soja*) and their expression analyses under drought condition

**DOI:** 10.1371/journal.pone.0283917

**Published:** 2023-04-19

**Authors:** The PLOS ONE Editors

The *PLOS ONE* Editors retract this article [1] because it was identified as one of a series of submissions for which we have concerns about authorship, competing interests, and peer review. We regret that the issues were not addressed prior to the article’s publication.

SUR, AI, and ZA did not agree with the retraction. GQ, MHNT, SF, FA, MSajjad, MShees, MU, and ZG either did not respond directly or could not be reached.
